# *N*-Cyclo­hexyl­nicotinamide

**DOI:** 10.1107/S1600536810023408

**Published:** 2010-06-23

**Authors:** Na Li

**Affiliations:** a Shenyang Research Institute of Chemical Industry, Shenyang 110021, People’s Republic of China

## Abstract

In the title compound, C_12_H_16_N_2_O, the dihedral angle between the pyridine ring and C/O/N plane is 22.93 (7)°. In the crystal structure, inter­molecular N—H⋯O hydrogen bonds link the mol­ecules, forming extended chains along [001]. π–π inter­actions between inversion-related pyridine rings [centroid–centroid distance = 3.825 (2)Å] are also observed.

## Related literature

For background information on metal-organic framework compounds, see: Subramanian & Zaworotko (1994 ▶); Kitagawa *et al.* (2004 ▶); Rosi *et al.* (2005 ▶). For details of the synthesis, see: Basolo *et al.* (2009 ▶).
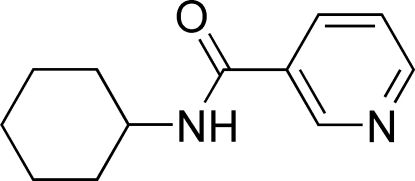


## Experimental

### 

#### Crystal data

C_12_H_16_N_2_O
*M* *_r_* = 204.27
Monoclinic, 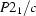

*a* = 17.596 (2) Å
*b* = 6.4050 (8) Å
*c* = 10.1167 (12) Å
β = 103.921 (2)°
*V* = 1106.7 (2) Å^3^
*Z* = 4
Mo *K*α radiation
μ = 0.08 mm^−1^
*T* = 296 K
0.32 × 0.30 × 0.22 mm

#### Data collection

Bruker APEXII CCD area-detector diffractometer
Absorption correction: multi-scan (*SADABS*; Sheldrick, 1996 ▶) *T*_min_ = 0.869, *T*_max_ = 1.000
5389 measured reflections
1956 independent reflections
1661 reflections with *I* > 2σ(*I*)
*R*_int_ = 0.013

#### Refinement

*R*[*F* ^2^ > 2σ(*F* ^2^)] = 0.036
*wR*(*F* ^2^) = 0.096
*S* = 1.06
1956 reflections
136 parameters
H-atom parameters constrained
Δρ_max_ = 0.10 e Å^−3^
Δρ_min_ = −0.17 e Å^−3^

### 

Data collection: *APEX2* (Bruker, 2003 ▶); cell refinement: *SAINT* (Bruker, 2003 ▶); data reduction: *SAINT*; program(s) used to solve structure: *SHELXS97* (Sheldrick, 2008 ▶); program(s) used to refine structure: *SHELXL97* (Sheldrick, 2008 ▶); molecular graphics: *SHELXTL* (Sheldrick, 2008 ▶); software used to prepare material for publication: *SHELXTL*.

## Supplementary Material

Crystal structure: contains datablocks global, I. DOI: 10.1107/S1600536810023408/pk2247sup1.cif

Structure factors: contains datablocks I. DOI: 10.1107/S1600536810023408/pk2247Isup2.hkl

Additional supplementary materials:  crystallographic information; 3D view; checkCIF report

## Figures and Tables

**Table 1:** Hydrogen-bond geometry (Å, °)

*D*—H⋯*A*	*D*—H	H⋯*A*	*D*⋯*A*	*D*—H⋯*A*
N2—H2⋯O1^i^	0.86	2.17	2.9998 (13)	162

Symmetry code: (i) 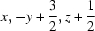.

**Fig. 1.**
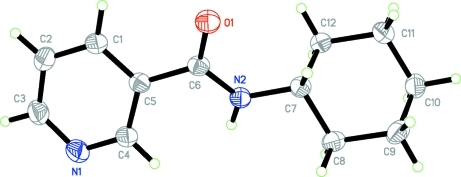
The molecular structure of the title compound showing 30% probability ellipsoids.

**Fig. 2.**
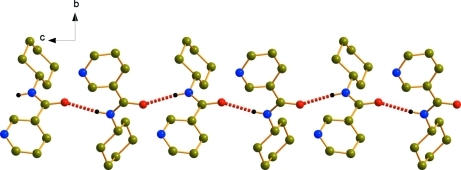
The one-dimensional chain structure of the title compound, showing N—H···O hydrogen bonds as red dashed lines.

## supplementary crystallographic information

### Comment

Metal-organic frameworks (MOFs) have attracted much attention because of their intriguing topologies (Subramanian & Zaworotko,1994; Kitagawa *et al.*, 2004; Rosi *et al.*, 2005). During our efforts to investigate the assembly of metal-organic coordination frameworks, a new compound was generated accidentally and its crystal structure is described in this paper.  A dedicated synthesis of the compound was previously described by Basolo *et al.*, (2009). The molecular structure of compound is shown in Fig. 1.  The dihedral angle between the mean plane of the pyridine ring and the plane formed by atoms C/O/N is 22.93 (7)°.  In the crystal structure N—H···O hydrogen bonds involving the acyl O atoms and the adjacent N—H group, form one-dimensional chains along [001] (Fig. 2). There are also π-π interactions involving inversion related pyridine rings.

### Experimental

All the starting materials and solvents for syntheses were obtained commercially and used as received. Zn(OAc)_2_.4H_2_O (21.8 mg, 0.1 mmol) and *N*-cyclohexylnicotinamide (20.4 mg, 0.1 mmol) were mixed in a CH_3_CN/H_2_O (20 ml, 1:1 *v*/*v*) solution with vigorous stirring for *ca* 30 min. The resulting solution was filtered and left to stand at room temperature. Pale-yellow prismatic crystals suitable for X-ray analysis were obtained by slow evaporation of the solvent over a period of 1 week.

### Refinement

Although all H atoms were visible in difference maps, they were placed in geometrically calculated positions, with C—H distances in the range 0.93–0.97Å and N—H distances of 0.86 Å, and included in the final refinement in the riding model approximation,with *U*_iso_(H) = 1.2*U*_eq_(C,*N*) for cyclohexyl and nicotinamide H atoms.

### Crystal data

**Table:**

C_12_H_16_N_2_O	*F*(000) = 440
*M**_r_* = 204.27	*D*_x_ = 1.226 Mg m^−^^3^
Monoclinic, *P*2_1_/*c*	Mo *K*α radiation, λ = 0.71073 Å
Hall symbol: -P 2ybc	Cell parameters from 2901 reflections
*a* = 17.596 (2) Å	θ = 2.8–29.5°
*b* = 6.4050 (8) Å	µ = 0.08 mm^−^^1^
*c* = 10.1167 (12) Å	*T* = 296 K
β = 103.921 (2)°	Block, pale yellow
*V* = 1106.7 (2) Å^3^	0.32 × 0.30 × 0.22 mm
*Z* = 4	

### Data collection

**Table:**

Bruker APEXII CCD area-detector diffractometer	1956 independent reflections
Radiation source: fine-focus sealed tube	1661 reflections with *I* > 2σ(*I*)
graphite	*R*_int_ = 0.013
φ and ω scans	θ_max_ = 25.0°, θ_min_ = 2.4°
Absorption correction: multi-scan (*SADABS*; Sheldrick, 1996)	*h* = −20→20
*T*_min_ = 0.869, *T*_max_ = 1.000	*k* = −7→7
5389 measured reflections	*l* = −7→12

### Refinement

**Table:**

Refinement on *F*^2^	Primary atom site location: structure-invariant direct methods
Least-squares matrix: full	Secondary atom site location: difference Fourier map
*R*[*F*^2^ > 2σ(*F*^2^)] = 0.036	Hydrogen site location: inferred from neighbouring sites
*wR*(*F*^2^) = 0.096	H-atom parameters constrained
*S* = 1.06	*w* = 1/[σ^2^(*F*_o_^2^) + (0.0474*P*)^2^ + 0.1872*P*] where *P* = (*F*_o_^2^ + 2*F*_c_^2^)/3
1956 reflections	(Δ/σ)_max_ < 0.001
136 parameters	Δρ_max_ = 0.10 e Å^−^^3^
0 restraints	Δρ_min_ = −0.16 e Å^−^^3^

### Special details

**Table:**

Geometry. All e.s.d.'s (except the e.s.d. in the dihedral angle between two l.s. planes) are estimated using the full covariance matrix. The cell e.s.d.'s are taken into account individually in the estimation of e.s.d.'s in distances, angles and torsion angles; correlations between e.s.d.'s in cell parameters are only used when they are defined by crystal symmetry. An approximate (isotropic) treatment of cell e.s.d.'s is used for estimating e.s.d.'s involving l.s. planes.
Refinement. Refinement of *F*^2^ against ALL reflections. The weighted *R*-factor *wR* and goodness of fit *S* are based on *F*^2^, conventional *R*-factors *R* are based on *F*, with *F* set to zero for negative *F*^2^. The threshold expression of *F*^2^ > σ(*F*^2^) is used only for calculating *R*-factors(gt) *etc*. and is not relevant to the choice of reflections for refinement. *R*-factors based on *F*^2^ are statistically about twice as large as those based on *F*, and *R*- factors based on ALL data will be even larger.

### Fractional atomic coordinates and isotropic or equivalent isotropic displacement parameters (Å^2^)

**Table:**

	*x*	*y*	*z*	*U*_iso_*/*U*_eq_	
O1	0.26804 (6)	0.73912 (16)	0.77836 (8)	0.0572 (3)	
N1	0.43960 (7)	1.0508 (2)	1.16302 (11)	0.0604 (3)	
N2	0.25623 (6)	0.64514 (17)	0.98679 (9)	0.0421 (3)	
H2	0.2706	0.6688	1.0729	0.051*	
C1	0.36028 (8)	1.0899 (2)	0.89337 (13)	0.0535 (4)	
H1	0.3346	1.1024	0.8019	0.064*	
C2	0.41240 (9)	1.2403 (2)	0.95508 (15)	0.0620 (4)	
H2A	0.4222	1.3564	0.9065	0.074*	
C3	0.44952 (8)	1.2162 (2)	1.08908 (15)	0.0597 (4)	
H3	0.4835	1.3208	1.1310	0.072*	
C4	0.38905 (8)	0.9069 (2)	1.10174 (13)	0.0500 (3)	
H4	0.3819	0.7899	1.1518	0.060*	
C5	0.34619 (7)	0.9201 (2)	0.96774 (11)	0.0401 (3)	
C6	0.28717 (7)	0.7604 (2)	0.90309 (11)	0.0408 (3)	
C7	0.19904 (7)	0.48073 (19)	0.93833 (11)	0.0402 (3)	
H7	0.2126	0.4131	0.8602	0.048*	
C8	0.20335 (8)	0.3175 (2)	1.04845 (13)	0.0479 (3)	
H8A	0.1929	0.3827	1.1288	0.057*	
H8B	0.2557	0.2590	1.0732	0.057*	
C9	0.14451 (9)	0.1438 (2)	1.00039 (15)	0.0572 (4)	
H9A	0.1584	0.0687	0.9263	0.069*	
H9B	0.1464	0.0462	1.0744	0.069*	
C10	0.06238 (9)	0.2285 (2)	0.95261 (14)	0.0556 (4)	
H10A	0.0269	0.1151	0.9166	0.067*	
H10B	0.0460	0.2884	1.0294	0.067*	
C11	0.05771 (8)	0.3929 (2)	0.84404 (13)	0.0543 (4)	
H11A	0.0053	0.4511	0.8203	0.065*	
H11B	0.0677	0.3288	0.7630	0.065*	
C12	0.11653 (7)	0.5674 (2)	0.89148 (13)	0.0482 (3)	
H12A	0.1144	0.6650	0.8174	0.058*	
H12B	0.1030	0.6423	0.9659	0.058*	

### Atomic displacement parameters (Å^2^)

**Table:**

	*U*^11^	*U*^22^	*U*^33^	*U*^12^	*U*^13^	*U*^23^
O1	0.0711 (6)	0.0719 (7)	0.0293 (5)	−0.0167 (5)	0.0134 (4)	−0.0038 (4)
N1	0.0581 (7)	0.0738 (8)	0.0439 (6)	−0.0118 (6)	0.0014 (5)	−0.0022 (6)
N2	0.0492 (6)	0.0490 (6)	0.0286 (5)	−0.0060 (5)	0.0102 (4)	−0.0030 (4)
C1	0.0543 (8)	0.0661 (9)	0.0378 (7)	−0.0094 (7)	0.0069 (6)	0.0063 (6)
C2	0.0659 (9)	0.0628 (9)	0.0563 (9)	−0.0178 (7)	0.0128 (7)	0.0086 (7)
C3	0.0556 (8)	0.0653 (9)	0.0554 (8)	−0.0164 (7)	0.0079 (7)	−0.0062 (7)
C4	0.0512 (7)	0.0587 (8)	0.0381 (7)	−0.0055 (6)	0.0068 (5)	0.0033 (6)
C5	0.0386 (6)	0.0502 (7)	0.0332 (6)	0.0011 (5)	0.0120 (5)	−0.0004 (5)
C6	0.0429 (7)	0.0490 (7)	0.0316 (6)	0.0017 (5)	0.0110 (5)	−0.0005 (5)
C7	0.0487 (7)	0.0405 (6)	0.0323 (6)	−0.0020 (5)	0.0111 (5)	−0.0045 (5)
C8	0.0562 (8)	0.0439 (7)	0.0421 (7)	0.0049 (6)	0.0090 (6)	0.0033 (6)
C9	0.0818 (10)	0.0390 (7)	0.0517 (8)	−0.0031 (7)	0.0176 (7)	0.0036 (6)
C10	0.0653 (9)	0.0554 (8)	0.0466 (8)	−0.0180 (7)	0.0144 (6)	−0.0041 (6)
C11	0.0544 (8)	0.0589 (9)	0.0455 (7)	−0.0084 (6)	0.0039 (6)	−0.0002 (6)
C12	0.0524 (7)	0.0430 (7)	0.0459 (7)	−0.0009 (6)	0.0054 (6)	0.0047 (6)

### Geometric parameters (Å, °)

**Table:**

O1—C6	1.2328 (14)	C7—C12	1.5196 (17)
N1—C4	1.3262 (17)	C7—H7	0.9800
N1—C3	1.3326 (19)	C8—C9	1.5176 (19)
N2—C6	1.3341 (15)	C8—H8A	0.9700
N2—C7	1.4576 (15)	C8—H8B	0.9700
N2—H2	0.8600	C9—C10	1.510 (2)
C1—C2	1.373 (2)	C9—H9A	0.9700
C1—C5	1.3783 (18)	C9—H9B	0.9700
C1—H1	0.9300	C10—C11	1.5096 (19)
C2—C3	1.365 (2)	C10—H10A	0.9700
C2—H2A	0.9300	C10—H10B	0.9700
C3—H3	0.9300	C11—C12	1.5203 (18)
C4—C5	1.3863 (17)	C11—H11A	0.9700
C4—H4	0.9300	C11—H11B	0.9700
C5—C6	1.4919 (17)	C12—H12A	0.9700
C7—C8	1.5164 (17)	C12—H12B	0.9700
			
C4—N1—C3	116.98 (11)	C7—C8—H8A	109.4
C6—N2—C7	122.71 (9)	C9—C8—H8A	109.4
C6—N2—H2	118.6	C7—C8—H8B	109.4
C7—N2—H2	118.6	C9—C8—H8B	109.4
C2—C1—C5	119.59 (12)	H8A—C8—H8B	108.0
C2—C1—H1	120.2	C10—C9—C8	111.48 (11)
C5—C1—H1	120.2	C10—C9—H9A	109.3
C3—C2—C1	118.70 (14)	C8—C9—H9A	109.3
C3—C2—H2A	120.6	C10—C9—H9B	109.3
C1—C2—H2A	120.6	C8—C9—H9B	109.3
N1—C3—C2	123.48 (13)	H9A—C9—H9B	108.0
N1—C3—H3	118.3	C11—C10—C9	111.33 (12)
C2—C3—H3	118.3	C11—C10—H10A	109.4
N1—C4—C5	124.11 (13)	C9—C10—H10A	109.4
N1—C4—H4	117.9	C11—C10—H10B	109.4
C5—C4—H4	117.9	C9—C10—H10B	109.4
C1—C5—C4	117.05 (12)	H10A—C10—H10B	108.0
C1—C5—C6	119.96 (11)	C10—C11—C12	111.68 (10)
C4—C5—C6	122.99 (11)	C10—C11—H11A	109.3
O1—C6—N2	122.41 (11)	C12—C11—H11A	109.3
O1—C6—C5	120.95 (11)	C10—C11—H11B	109.3
N2—C6—C5	116.64 (10)	C12—C11—H11B	109.3
N2—C7—C8	110.04 (9)	H11A—C11—H11B	107.9
N2—C7—C12	111.81 (10)	C7—C12—C11	110.90 (11)
C8—C7—C12	110.86 (10)	C7—C12—H12A	109.5
N2—C7—H7	108.0	C11—C12—H12A	109.5
C8—C7—H7	108.0	C7—C12—H12B	109.5
C12—C7—H7	108.0	C11—C12—H12B	109.5
C7—C8—C9	111.12 (10)	H12A—C12—H12B	108.0
			
C5—C1—C2—C3	0.5 (2)	C1—C5—C6—N2	−157.12 (12)
C4—N1—C3—C2	−1.9 (2)	C4—C5—C6—N2	23.14 (18)
C1—C2—C3—N1	2.0 (3)	C6—N2—C7—C8	153.38 (11)
C3—N1—C4—C5	−0.6 (2)	C6—N2—C7—C12	−82.96 (14)
C2—C1—C5—C4	−2.8 (2)	N2—C7—C8—C9	−179.80 (10)
C2—C1—C5—C6	177.44 (12)	C12—C7—C8—C9	55.99 (14)
N1—C4—C5—C1	3.0 (2)	C7—C8—C9—C10	−55.68 (15)
N1—C4—C5—C6	−177.30 (12)	C8—C9—C10—C11	54.95 (15)
C7—N2—C6—O1	1.82 (19)	C9—C10—C11—C12	−54.88 (16)
C7—N2—C6—C5	−178.83 (10)	N2—C7—C12—C11	−178.89 (10)
C1—C5—C6—O1	22.24 (18)	C8—C7—C12—C11	−55.69 (14)
C4—C5—C6—O1	−157.50 (13)	C10—C11—C12—C7	55.31 (15)

### Hydrogen-bond geometry (Å, °)

**Table:**

*D*—H···*A*	*D*—H	H···*A*	*D*···*A*	*D*—H···*A*
N2—H2···O1^i^	0.86	2.17	2.9998 (13)	162

Symmetry codes: (i) *x*, −*y*+3/2, *z*+1/2.

## References

[bb1] Basolo, L., Beccalli, E. M., Borsini, E. & Broggini, G. (2009). *Tetrahedron*, **65**, 3486–3490.

[bb2] Bruker (2003). *APEX2* and *SAINT* Bruker AXS Inc., Madison, Wisconsin, USA.

[bb3] Kitagawa, S., Kitaura, T. & Noro, S. (2004). *Angew. Chem. Int. Ed.* **43**, 2334–2375. 10.1002/anie.200300610 15114565

[bb4] Rosi, N. L., Kim, J., Eddaoudi, M., Chen, B., O’Keeffe, M. & Yaghi, O. M. (2005). *J. Am. Chem. Soc.* **127**, 1504–1518. 10.1021/ja045123o 15686384

[bb5] Sheldrick, G. M. (1996). *SADABS* University of Göttingen, Germany.

[bb6] Sheldrick, G. M. (2008). *Acta Cryst.* A**64**, 112–122. 10.1107/S0108767307043930 18156677

[bb7] Subramanian, S. & Zaworotko, M. J. (1994). *Coord. Chem. Rev.* **137**, 357–401.

